# Best Practices in the Clinical Management of Progressive Supranuclear Palsy and Corticobasal Syndrome: A Consensus Statement of the CurePSP Centers of Care

**DOI:** 10.3389/fneur.2021.694872

**Published:** 2021-07-01

**Authors:** Brent Bluett, Alexander Y. Pantelyat, Irene Litvan, Farwa Ali, Diana Apetauerova, Danny Bega, Lisa Bloom, James Bower, Adam L. Boxer, Marian L. Dale, Rohit Dhall, Antoine Duquette, Hubert H. Fernandez, Jori E. Fleisher, Murray Grossman, Michael Howell, Diana R. Kerwin, Julie Leegwater-Kim, Christiane Lepage, Peter Alexander Ljubenkov, Martina Mancini, Nikolaus R. McFarland, Paolo Moretti, Erica Myrick, Pritika Patel, Laura S. Plummer, Federico Rodriguez-Porcel, Julio Rojas, Christos Sidiropoulos, Miriam Sklerov, Leonard L. Sokol, Paul J. Tuite, Lawren VandeVrede, Jennifer Wilhelm, Anne-Marie A. Wills, Tao Xie, Lawrence I. Golbe

**Affiliations:** ^1^Neurology, Pacific Central Coast Health Center, Dignity Health, San Luis Obispo, CA, United States; ^2^Neurology, Stanford University, Stanford, CA, United States; ^3^Neurology, The Johns Hopkins Hospital, Johns Hopkins Medicine, Baltimore, MD, United States; ^4^Neurology, University of California, San Diego, San Diego, CA, United States; ^5^Neurology, Mayo Clinic, Rochester, MN, United States; ^6^Neurology, Lahey Hospital and Medical Center, Burlington, MA, United States; ^7^Ken and Ruth Davee Department of Neurology, Feinberg School of Medicine, Northwestern University, Chicago, IL, United States; ^8^Neurology, Surgery, University of Chicago, Chicago, IL, United States; ^9^Neurology, University of California, San Francisco, San Francisco, CA, United States; ^10^Neurology, Oregon Health and Science University, Portland, OR, United States; ^11^Neurology, University of Arkansas for Medical Sciences, Little Rock, AK, United States; ^12^Service de Neurologie, Département de Médecine, Unité de Troubles du Mouvement André-Barbeau, Centre Hospitalier de l'Université de Service de Neurologie, Centre Hospitalier de l'Université de Montréal (CHUM), Montreal, QC, Canada; ^13^Center for Neurological Restoration, Neurological Institute, Cleveland Clinic, Cleveland, OH, United States; ^14^Neurological Sciences, Rush Medical College, Rush University, Chicago, IL, United States; ^15^Neurology, University of Pennsylvania, Philadelphia, PA, United States; ^16^Neurology, Medical School, University of Minnesota, Minneapolis, MN, United States; ^17^Geriatrics, Presbyterian Hospital of Dallas, Dallas, TX, United States; ^18^Neurology, College of Medicine, University of Florida, Gainesville, FL, United States; ^19^Neurology, The University of Utah, Salt Lake City, UT, United States; ^20^Neurology, Massachusetts General Hospital and Harvard Medical School, Boston, MA, United States; ^21^Neurology, Medical University of South Carolina, Charleston, SC, United States; ^22^Neurology, Michigan State University, East Lansing, MI, United States; ^23^Neurology, School of Medicine, University of North Carolina at Chapel Hill, Chapel Hill, NC, United States; ^24^Neurology, School of Medicine, University of California, San Francisco, San Francisco, CA, United States; ^25^Neurology, Rutgers Robert Wood Johnson Medical School, New Brunswick, NJ, United States

**Keywords:** progressive supranuclear palsy, corticobasal syndrome, management, consensus, CurePSP, treatment, symptomatic, palliative

## Abstract

Progressive supranuclear palsy (PSP) and corticobasal syndrome (CBS; the most common phenotype of corticobasal degeneration) are tauopathies with a relentless course, usually starting in the mid-60s and leading to death after an average of 7 years. There is as yet no specific or disease-modifying treatment. Clinical deficits in PSP are numerous, involve the entire neuraxis, and present as several discrete phenotypes. They center on rigidity, bradykinesia, postural instability, gait freezing, supranuclear ocular motor impairment, dysarthria, dysphagia, incontinence, sleep disorders, frontal cognitive dysfunction, and a variety of behavioral changes. CBS presents with prominent and usually asymmetric dystonia, apraxia, myoclonus, pyramidal signs, and cortical sensory loss. The symptoms and deficits of PSP and CBS are amenable to a variety of treatment strategies but most physicians, including many neurologists, are reluctant to care for patients with these conditions because of unfamiliarity with their multiplicity of interacting symptoms and deficits. CurePSP, the organization devoted to support, research, and education for PSP and CBS, created its CurePSP Centers of Care network in North America in 2017 to improve patient access to clinical expertise and develop collaborations. The directors of the 25 centers have created this consensus document outlining best practices in the management of PSP and CBS. They formed a writing committee for each of 12 sub-topics. A 4-member Steering Committee collated and edited the contributions. The result was returned to the entire cohort of authors for further comments, which were considered for incorporation by the Steering Committee. The authors hope that this publication will serve as a convenient guide for all clinicians caring for patients with PSP and CBS and that it will improve care for patients with these devastating but manageable disorders.

## Introduction

Neurologists and other health professionals at the 25 sites in the US and Canada comprising the CurePSP Centers of Care (CoC) have collaborated to produce this “best practices” document on the therapeutic management of progressive supranuclear palsy (PSP) and corticobasal syndrome (CBS). CurePSP is a lay-led organization founded in 1990 to sponsor research in PSP and CBD and to provide education and support for those with PSP and CBD. CurePSP established the CoC network in 2017 with plans to expand to 40 sites in North America and Europe by 2022. The CoCs are dedicated to providing state-of-the-art clinical care to patients with PSP and CBS, to educate other professionals both locally and globally, and to collaborate in discovering ways to improve that care.

A Steering Committee comprising authors B. Bluett, L. Golbe, I. Litvan, and A. Pantelyat divided the subject into 12 areas and formed writing committees from the Centers of Care site directors, all neurologists specializing in movement disorders or behavioral neurology. The Steering Committee recruited one member of each writing committee to serve as its chair. The 22 site directors recruited other clinicians from their sites as necessary to assist in the writing. The Steering Committee then edited the 12 resulting sections of text into a unified document, submitted it to the other authors for review and comment and made final revisions as appropriate.

Driving this effort is our observation that most neurologists are uncomfortable managing patients with PSP and CBS and refer them to movement disorder specialists, who themselves may feel that little can be done. While treatment of PSP and CBS remains symptomatic, much can in fact be offered to ease both physical and emotional suffering and by avoiding complications, to delay disability and perhaps death.

The review discusses clinical pathophysiology to the extent appropriate to our goal of conveying management advice. It does not discuss diagnosis, a rapidly changing area that deserves its own “best practices” review. Throughout, we use the term CBS rather than corticobasal degeneration (CBD), a pathologically defined entity very difficult to diagnose during life. As will become clear in many parts of this review, there have been few randomized, blinded trials of symptomatic treatment in PSP or CBS. Most of our management recommendations, therefore, rely on personal experience, small or retrospective series, and extrapolation from trials in Parkinson's disease or other better-studied conditions. We emphasize the need to assess patients' medications regularly for effectiveness and toxicity over the course of treatment. In our experience, medications for dementia, motor Parkinsonism, and bladder dysfunction are especially likely to be continued past the limit of their utility.

## Parkinsonism

The motor symptoms of PSP may include Parkinsonism with axial greater than appendicular rigidity, bradykinesia/hypokinesia, tremor, gait difficulty, gait freezing, and loss of balance with frequent falls, although all of these vary significantly across subtypes (1–3) (Table 1). No disease-modifying therapies for PSP are available to date. However, some therapeutic interventions may improve bradykinesia, rigidity, and tremor in PSP.

**Table 1:** Management of motor Parkinsonism in PSP/CBS.

1. Levodopa trial
a. Helpful only when bradykinesia, rigidity or tremor affects daily activities.
b. Titrate carbidopa (or equivalent)/levodopa 25/100 over at least 1 month to 900–1,200 mg per day.
c. Maintain maximum tolerated dosage for at least 1 month.
d. If no benefit, or if adverse effects (including dyskinesias) occur, gradually taper over at least 2 weeks and try to discontinue.
2. Ancillary dopaminergic medications are unnecessary and should generally be avoided
a. Avoid “controlled-release” and “extended-release” preparations
b. Avoid dopamine receptor agonists
c. Avoid monoamine oxidase B inhibitors
d. Avoid catecholamine O-methyltransferase inhibitors
e. Avoid adenosine A2A receptor antagonists
3. Non-dopaminergics are not recommended and pure anticholinergics should be avoided.

### Dopaminergic Medications in PSP

Levodopa (with a peripheral decarboxylase inhibitor such as carbidopa or benserazide) is the principal pharmacologic agent used for dopaminergic replacement therapy in PSP, with a more robust response generally seen in the PSP-Parkinsonism (PSP-P) subtype. Bradykinesia, rigidity, and tremor seen in any phenotype of PSP may respond as well as in PSP-P, but postural instability is unlikely to respond. Based on retrospective studies, 20–30% of pathologically confirmed and 20–40% of clinically diagnosed PSP patients reported a beneficial response to levodopa alone or in combination with another dopaminergic agent (such as amantadine) (4–9). These responses generally occur only early in the disease course and persist for only a few months. Furthermore, levodopa treatment should be attempted only in patients for whom rigidity, bradykinesia, or tremor are impairing daily activities. With those caveats, we recommend a trial of levodopa titrated over a period of at least 1 month up to 900–1,200 mg daily as tolerated. The patient should be observed for at least 1 month at this dosage. If ineffective, levodopa should then be gradually reduced to determine the optimal dosage and if there is no benefit, tapered over at least 2 weeks and discontinued. During the levodopa taper, the patient may exhibit a sudden worsening, in which case levodopa should be maintained at that optimal dosage. At a later date, another attempt to taper can be initiated. Studies of dopaminergic agents aside from levodopa have shown minimal to no benefit in PSP (10–14).

### Non-dopaminergic Medications for Parkinsonism in PSP

Donepezil was found to worsen motor symptoms while improving cognitive function in PSP patients in a randomized, double-blind, placebo-controlled crossover trial (15). Despite several retrospective reports of clinically meaningful improvement in motor function on low doses of amitriptyline (7, 9, 16), we avoid this medication in PSP because of its anticholinergic side effects and risk of exacerbating falling.

Coenzyme Q-10 in its liposomal formulation at a dosage of 100 mg tid gave modest but statistically significant benefit in the PSP Rating Scale, mostly in the gait and balance items, in a 6-week double-blind trial (17). A 12-month double-blind trial of the non-liposomal formulation of Coenzyme Q-10 at a dosage of 2,400 mg per day produced a favorable trend, but the high dropout rate reduced the power of the study and the result was non-significant (*p* = 0.07) (18).

A double-blind, placebo-controlled crossover study in 10 PSP patients receiving single doses of zolpidem, carbidopa/levodopa, or placebo showed no difference between carbidopa/levodopa and placebo, but patients on zolpidem 5 mg showed a 6.5% improvement in the UPDRS-III over baseline, a statistically significant result (19). However, that result was driven by major improvement in only 2 of the 10 patients, zolpidem 10 mg. gave no improvement, and somnolence and worsening of falls limit utility of zolpidem in this setting.

### Parkinsonism in CBS

Existing treatments of Parkinsonism in CBS have minimal to no lasting effects. In two small series of pathologically confirmed patients (total *n* = 20), approximately half reported transient, modest improvement (20, 21). Retrospective review in 147 clinically diagnosed patients found that the most effective agent appeared to be carbidopa-levodopa, with improvement of Parkinsonism in 20% of treated individuals. Dopamine agonists, amantadine, and selegiline were not effective (22, 23).

As dose-related fluctuations in dopaminergic response have never been clearly reported in PSP/CBS, there is no need for controlled-release or extended-release preparations or for monoamine oxidase B inhibitors or catecholamine O-methyltransferase inhibitors in this setting.

### Surgical Approaches

The effects of deep brain stimulation of the globus pallidus pars interna and subthalamic nucleus on Parkinsonism, gait difficulty, and balance in PSP have failed to show convincing benefit (24–29). Pedunculopontine nucleus DBS is still being investigated in PSP, but to date has shown modest and inconsistent benefit as well. Spinal cord stimulation for freezing of gait in PSP is under study. We do not recommend the procedure outside of a formal research protocol (30).

## Gait Dysfunction and Postural Instability

A central diagnostic feature of PSP is the early appearance of gait dysfunction and postural instability, resulting in increasing falls (31, 32) (Table 2). Relative to other Parkinsonian syndromes, gait dysfunction in PSP is characterized by step asymmetry and large lateral deviations and may be described as stiff, clumsy, and lurching “like a drunken sailor” (33). In contrast to the *en bloc* turning typical of Parkinson's disease, PSP patients may pivot carelessly and often retropulse, with a risk of falling. Falls tend to begin within the first 3 years of symptom onset and their sudden and unpredictable nature makes them difficult to prevent. Falls may lead to significant injury including lacerations and fractures (34).

**Table 2:** Management of gait dysfunction in PSP/CBS.

1. Pharmacologic therapy
a. Amantadine
i. Start at 50–100 mg daily and titrate at intervals of at least 2 weeks
ii. Do not exceed 100 mg TID.
iii. Monitor for psychosis, confusion, and constipation.
iv. Inspect for livedo reticularis and reassure patient that it is benign.
b. Coenzyme Q-10
i. Modest benefit on average but some patients respond well
ii. Use liposomal form (a liquid) at 100 mg TID
iii. If no benefit after 2 months, discontinue.
2. Non-pharmacologic therapy
a. Physical therapy
i. Early intervention recommended
ii. Focused on postural stability and gait re-training
iii. Balance, eye movement training and visual awareness training may help.
b. Exercise
i. Aerobic exercise
1. If tolerated and safe
2. Obtain approval from PCP or cardiologist if cardiovascular history is present.
ii. Recumbent bicycle is recommended.
c. Assistive devices
i. Cane should be prescribed with caution
1. Tripping hazard, as patients tend to carry cane hanging from wrist
2. Fails to prevent falls in other directions.
ii. Weighted walker
iii. Wheelchair
iv. A lightweight transport wheelchair is useful when the caregiver cannot lift a standard wheelchair into a car's trunk.

### Causes of Gait and Postural Instability

Falls in PSP result from multiple factors including axial rigidity, bradykinesia, freezing of gait, reduced or absent postural reflexes, visual-vestibular deficits, impulsivity, and decreased insight (9, 31, 33). PSP patients' fall risk may be assayed at a clinic visit by careful observation of turning. Those characterized as frequent fallers have been found to have disproportionately poor performance on turning and pivoting, which can be evaluated clinically to identify those at greatest risk (32).

Impaired multisensory integration leads to maladaptive motor corrections in response to perturbations of the center of gravity (35). The tilting platform in the laboratory may mimic real-life backward perturbations, and the abnormal toe push-off exacerbates backward postural instability (35). Persons with PSP may also have increased postural sway when standing quietly without perturbation.

Abnormal peripheral vestibular, ocular motor, and cognitive deficits also exacerbate postural instability in PSP. Translational vestibulo-ocular reflexes are diminished with near target viewing (36). Difficulty infraducting the eyes limits the ability to detect surface changes on the ground such as impending curbs and convergence insufficiency limits depth perception. Many patients with PSP also have poor insight and impulsivity, likely due to fronto-subcortical dysfunction (37). Freezing of gait is common early in the course of most PSP phenotypes and may increase over time. Freezing is associated with speech impairment, suggesting they may have similar underlying pathophysiology and potentially similar treatment approaches (for example, rhythmic auditory stimulation) (38).

### Evaluation of Gait and Balance

Evaluation of multi-directional walking, turning and navigating obstacles is critical as deficits in visual fixation, visual scanning and motor planning impair ability to complete more complex movements (39). The Berg Balance test and the Mini-BESTest have been used safely and productively in PSP to evaluate gait and balance. Therapists typically examine posterior stability with the pull test or backward compensatory stepping in addition to assessing static balance. Examination should also include sit-to-stand, which may reveal altered sequencing due to lack of trunk flexion and anterior weight shift. Maintaining a seated position may be precarious due to imbalance, poor visuo-spatial skills, and truncal rigidity in extension. Ill-advised, impulsive arising from a chair is a frequent source of falls (the “rocket sign of PSP”) (40). Returning to the seat may be impaired by deficits in hip and knee flexion, motor sequencing, depth perception, downgaze, and impulse control (41).

### Pharmacological Treatment of Gait Dysfunction

There are limited data available on pharmacological treatment of gait dysfunction in PSP. Poor or absent response of gait impairment or freezing to levodopa or other dopaminergic agents is typical (9, 42–44). Variable effects have been reported for amantadine, an NMDA-receptor antagonist (9, 10, 45). In our experience, amantadine may improve gait and freezing. We recommend at most 100 mg TID to minimize adverse events such as delirium, visual hallucinations, livedo reticularis, or peripheral edema. Amantadine should be started at 50–100 mg once daily and titrated at intervals of at least 2 weeks. In our experience rasagiline may also improve FoG in PSP, although it is less effective than amantadine. The recommended dosage is 0.5 mg daily for 1 week, then 1 mg daily.

Amitriptyline and the centrally acting “pure anticholinergic” medications such as benztropine and trihexyphenidyl may worsen gait in PSP and should be avoided (7, 46–50). A 2-month trial of liposomal coenzyme Q-10 100 mg (1 ml) TID is also recommended. While only a few patients will respond, the magnitude of the response can be highly useful and adverse effects are minimal (17, 18).

### Non-pharmacologic Therapy for Gait and Postural Instability

Early intervention by physical therapy is recommended to improve gait and balance and avoid falls. Interventions that address visual impairment (including gaze shifting and eye movement training) may provide additional benefit when coupled with gait and balance therapy (39). A recent systematic review of exercise and physical activity for PSP identified 11 studies but noted limitations in methodology and understanding of the effects of structured physical activity in PSP (51). Body-weight-supported treadmill training, robot-assisted gait training, and music-cued movement training may be beneficial early in PSP to improve gait and balance (51). There is insufficient evidence regarding the benefits of therapeutic exercise in more advanced PSP.

Early in the disease process, physical therapists often introduce the idea of an assistive device. However, a cane reduces falls only in one direction and can become a tripping hazard because of visual scanning deficits, impulsivity, and impaired postural responses. Furthermore, many patients merely carry the cane hanging from an arm, exacerbating the tripping hazard. It is critical to educate the patient and caregiver regarding the importance of fall prevention and of the need to remain proactive in this regard. As soon as balance assessment by physical therapy advises ambulatory support, a weighted walker should be prescribed to prevent falls and injuries. If physical therapy evaluation is not readily available, a walker should be prescribed at the onset of the need for even intermittent external balance support. Since PSP patients have a tendency for posterior falls, a heavier walker with a wide base and reverse braking mechanism such as the U-Step 2 walker (In-Step Mobility Products, Skokie, IL, USA), is preferred over a lightweight rollator or standard frame rolling walker. As community mobility becomes more challenging and less safe, transport wheelchairs are an excellent option for continued community level participation.

The decision to transition to wheelchair mobility is not always clear and requires shared decision making with the patient and care partner. Once transition to a wheelchair is agreed upon, a referral to a wheelchair clinic is optimal for appropriate wheelchair prescription. A lightweight wheelchair with a waterproof seat is recommended to maximize community participation and safe mobility and to minimize caregiver burden. Tilt-in-space wheelchairs should be considered for positioning to prevent patients from falling out and to allow for optimal swallowing, pressure relief, and compensation for lack of downward gaze. Power mobility is often not recommended in PSP due to poor visual scanning and impulsivity but may work well in carefully selected patients.

### Role of Exercise in Gait and Balance

Few studies have addressed the efficacy of aerobic exercise in PSP and CBS. Case reports in PSP incorporating aerobic exercise have demonstrated improvement in balance and falls frequency (52), improved ambulation endurance (53), and walking distance (54). The advice and consent of the patient's primary care provider or cardiologist should precede the aerobic regimen. While a review of physical rehabilitation in PSP found possible improvements in balance and gait (55), a separate review of structured aerobic exercise found the improvement in balance and timed walking measures to be statistically non-significant (51). Despite the paucity of data, aerobic exercise is worth an empirical trial in PSP to augment balance, reduce falls, and improve conditioning. Experience shows a recumbent bicycle is a good option for home aerobic exercise because of its safety.

## Dystonia

### Oral Medications

Oral medications are a mainstay in treatment for symptomatic relief of dystonia, though evidence for their efficacy in PSP and CBS is limited to anecdotal experience (56) (Table 3). Muscle relaxants such as baclofen are primarily used in spasticity treatment (57). Although no controlled studies have evaluated their efficacy specifically for dystonia (58), they may have efficacy for this indication in PSP and CBS in certain cases. Dosing starts at 5 mg once daily and generally titrates to 10 mg TID. Caution should be used in patients with marginal gait function, as baclofen may cause weakness, orthostatic hypotension, sedation, or confusion. Treatment with benzodiazepines (most commonly clonazepam starting at 0.25 mg daily, titrating to efficacy, toxicity or 3 mg/day) may be attempted for dystonia in PSP and CBD (56, 57). Anticholinergics (e.g., trihexyphenidyl) may be used for dystonia of other causes, but should generally be avoided in PSP and CBS due to potential cognitive and gait impairment (57).

**Table 3:** Management of dystonia in PSP/CBS.

1. Pharmacologic therapy
a. Baclofen started at 5 mg daily, titrating gradually to no more than 10 mg TID
b. Clonazepam starting at 0.25 mg daily, titrating gradually to no more than 3 mg daily
c. Trihexyphenidyl not recommended due to central and peripheral anticholinergic side effects
d. BoNT injections are the most effective treatment for focal dystonia
e. Dystonia and spasticity in CBS may require higher BoNT doses than in other conditions.
2. Non-pharmacologic therapy
a. No formal studies demonstrate benefit of physical therapy for dystonia in PSP/CBS.
b. Occupational therapy may be beneficial for dystonia in PSP/CBS.
(i) Home health assessment
(ii) Evaluation for orthoses such as splints/braces
(iii) Exercises to optimize upper limb function

### Botulinum Toxin Injections

In our experience, botulinum toxin (BoNT) injections are the most effective treatment for focal dystonia, focal rigidity, and associated pain in PSP and CBS (58). However, studies of BoNT for limb and axial dystonia in PSP and CBS are limited to open-label series. In CBS patients with focal hand dystonia, BoNT may be helpful for muscle relaxation, ease of dressing and prevention of pain and palmar infections (59, 60). Dystonia in the setting of CBS is often mixed with spasticity and may require higher doses of BoNT than for dystonia or spasticity occurring in other disorders. Of note, functional improvement after injection for limb dystonia in PSP and CBS should not be expected.

For use in cervical dystonia, the American Academy of Neurology assigns Level A efficacy rating to abobotulinumtoxinA and rimabotulinumtoxinB; and Level B efficacy to onabotulinumtoxinA and incobotulinumtoxinA (61, 62). Dysphagia is a potential side effect of BoNT injections of the neck, occurring in up to 19% of cases after BoNT A injection and 48% after BoNT B injection (63). This may produce aspiration and its complications, especially in patients with existing dysphagia. With this important caveat, we recommend it in PSP and CBS primarily to reduce discomfort.

### Physical Therapy

The potential benefits of physical therapy for dystonia of any cause are not well-characterized (64). Optimal methods have not been systematically studied. Rather, individual therapists have promoted different strategies and techniques based on personal experience (65–68). There are no large-scale, controlled, blinded studies comparing strategies or demonstrating the efficacy of PT for dystonia and some of the most objective studies have failed to show benefit (65, 68). Whether this reflects the futility of PT or methodological issues remains unclear. The most recent systematic review of 45 publications found insufficient evidence to recommend any particular physical therapy strategy to treat dystonia in PSP or CBS (69). However, current literature has not sufficiently evaluated the efficacy of neuromodulation (i.e., transcranial direct current stimulation) combined with motor training (69).

### Occupational Therapy

The use of orthoses, devices such as splints and braces supporting an affected body part, has not been formally studied in PSP and CBS. Orthoses can be considered in conjunction with passive range of motion exercises and botulinum toxin injections to slow progression of contractures in PSP and CBS. PSP patients with retrocollis usually do not tolerate a rigid orthotic. Anterocollis can occur in PSP and may benefit from a hard or soft collar, assuming that a steroid-responsive extensor myopathy is not present. Striatal hand deformities, specifically swan neck deformities, may be alleviated with ring splints, which reduce abnormal hyperextension of the digits (70). Oromandibular dystonia is occasionally encountered in PSP. While dental splints have been reported to improve oromandibular dystonia in patients with Parkinson's disease and idiopathic dystonia (71), neither the literature nor our experience supports their use in PSP and CBS. A report of two CBS cases showed that treatment with videogame-based feedback appeared to improve pinch/grasp forces and apraxia but increased upper limb pain (72). Despite the absence of specific evidence, we recommend that affected individuals with PSP and CBS undergo a combination of three elements: OT that includes home health assessment, an evaluation for devices to support the affected body part, and exercises to optimize upper limb function.

## Eyelid and Visual Dysfunction

### Phenomenology and Examination

Ocular motor abnormalities in PSP are a hallmark of this disorder (Table 4). Decreased blink and tear production with resulting corneal drying and ocular irritation can contribute to blurred vision. Diplopia can occur in PSP/CBS due to convergence insufficiency or asymmetric duction impairment. The addition of prisms to glasses can help reading in select cases. However, their use is impractical in most patients due to rapid progression of saccadic slowing and hypometria.

**Table 4:** Management of eyelid and visual dysfunction in PSP/CBS.

1. Pharmacologic therapy
a. BoNT injections recommended for blepharospasm/lid levator inhibition
i. EMG guidance not necessary or recommended
ii. Pretarsal injections only recommended for refractory cases
b. Improve tear volume
i. Artificial tears
1. Glycerin
2. Carboxymethylcellulose
3. Polyethylene glycol
ii. Preservative-free lubricants
2. Non-pharmacologic therapy
a. Eyelid crutches
b. Sensory trick eyewear frames
c. Prevent conjunctival drying with humidifiers, warm wet compresses, avoiding forced air ventilation, and protective eyewear
d. Binocular prisms
i. For gaze limitation, not for diplopia
ii. Use with caution during ambulation
e. Environmental modifications
i. Elevate dinner plate
ii. Remove loose rugs, low coffee tables, and children's toys from floors
iii. Follow dinner fork or finger down to target
f. Referral to ophthalmologist or neuro-optometrist for consideration of:
i. Measures to reduce inflammation related to drying
ii. Improve tear retention

Examination of the eyes should start with inspection for subtle signs of inflammation such as redness of the eyelid margins (73), an early sign of conjunctival dryness or less frequently, infection. It should also include evaluation for reduced blink rate, tearing ability, and ability to reopen eyes after forceful closure (74, 75).

The examination continues with an evaluation of vertical and horizontal saccades, smooth pursuit, vergence and fixation. Optokinetic nystagmus (OKN) testing (typically with OKN strips) can help elicit subtle slowing, irregularity or loss of amplitude of vertical saccades, and can confirm an abnormality suspected on gross saccade testing. Saccadic abnormalities include a delay in initiation, need for a head thrust or a blink to initiate saccades, reduced velocity or range of excursion in vertical or horizontal meridians, and hypometria. Smooth pursuits may show a reduced range or velocity with saccadic intrusions. When there is slowing of saccades, convergence insufficiency is to be expected and is a frequent cause of diplopia. During fixation, square wave jerks may be noted and, although non-specific, in PSP these tend to be more frequent and larger than in other conditions (76) and the resulting oscillopsia may degrade visual performance.

It is important to remember that visual impairment in PSP/CBS patients may be related to age or to other comorbidities. Glaucoma, cataracts or macular degeneration are common in older patients. Examination of near visual acuity (with best refractive correction) provides a quick screen for any significant visual impairment. Confrontational visual field evaluation with double simultaneous stimulation is useful when highly lateralized pathology like CBD is suspected (77).

PSP/CBS patients commonly suffer from blepharospasm (abnormal contraction of the eyelid closing muscles) or lid levator inhibition (inability to initiate voluntary eyelid opening; “apraxia of eyelid opening”) (74, 77, 78).

### Management

Blepharospasm is typically unresponsive to systemic medication. The preferred treatment is BoNT injections into the orbital, preseptal and pretarsal segments of the orbicularis oculi muscle (62, 79). Only one open-label study has evaluated pretarsal BoNT injections in PSP. That favorable response and our own experience prompt us to recommend this approach for both lid levator inhibition and blepharospasm, which may coexist in the same patient (80). For blepharospasm (81), efficacy of types A and B is comparable (82–85). Electromyographic guidance is not necessary for these injections (86, 87). Pretarsal injections should be reserved for refractory cases because of the risk of ptosis and local bleeding.

Lid levator inhibition, like blepharospasm, is typically refractory to systemic pharmacologic treatment and we do not recommend oral pharmacotherapy for either blepharospasm or lid levator inhibition in PSP/CBS even when BoNT injections are ineffective.

Non-pharmacological treatment strategies for eyelid dysfunction include devices like eyelid crutches and sensory trick eyewear frames (88). Neuro-ophthalmologic and neuro-optometric consultation can be valuable for management of eyelid and visual dysfunction.

Decreased blink rate can produce conjunctival drying leading to dry eye, blepharitis and exposure keratitis with photophobia. Visual impairment may result from lid closure and exposure keratopathy (89). This can be treated conservatively with humidifiers, warm wet compresses, avoiding forced air ventilation and protective eyewear (89, 90). Tear volume can be improved by using over-the-counter artificial tear drops and preservative-free lubricants. Carboxymethylcellulose, which maintains the lipid layer of the tear film, may be more efficacious than artificial tears containing only glycerin (89, 91–93). Drops containing propylene glycol/polyethylene glycol may also provide benefit.

Ocular inflammation as a result of chronic exposure should be treated by an ophthalmologist with topical therapy like prednisone or cyclosporine (89, 91, 94, 95). Punctal plugs can be used to prevent outflow of tears but are usually effective for only a few months (90). Advanced devices to stimulate tear production should be discussed with an ophthalmologist (94).

For managing ocular movement impairment, combining eye movement exercises with balance training may improve gaze control (96). Occlusion may help in binocular diplopia, although saccadic impairment still typically limits the ability to read. A monocular prism does not typically help diplopia in PSP/CBS. Binocular prisms can help compensate for the effects of binocular range of gaze limitation, with modifications as the disease progresses. Bifocals and progressive lenses should not be used during ambulation as it may increase the risk of falls. Elevation of the dinner plate on a box or pursuing a fork downward from a position of primary gaze can improve visual performance when eating.

## Constipation and Urinary Symptoms

### Constipation

Constipation is common in PSP, affecting 71–80% of patients (97, 98) (Tables 5, 6). There are several putative causes of constipation in PSP including sedentary lifestyle, hypodipsia, central dysautonomia, and side effects of medications such as amantadine, anticholinergic agents, and opiates (99, 100). The consistency and frequency of bowel movements may be monitored during treatment using the Bristol stool chart, a clinical tool to evaluate colonic transit time (101).

**Table 5:** Management of constipation in PSP/CBS.

1. Non-pharmacologic therapy
a. Increased physical activity
b. Adequate hydration until suppertime to minimize nocturnal trips to void
c. Fiber supplementation
d. Minimize
i. Anticholinergics
ii. Amantadine
iii. Opiates
2. Pharmacologic therapy
a. Stool softener, docusate starting at 100 mg daily titrated weekly to 100 mg TID
b. Osmotic laxatives
i. Polyethylene glycol
ii. Magnesium citrate
iii. Lactulose
iv. OTC enema preparations of saline or other hyperosmolar salts
c. Stimulants for very short-term use
i. Bisacodyl
ii. Senna
d. Prescription medications for refractory cases
i. Linaclotide (guanylate cyclase C agonist)
ii. Prucalopride (selective 5-HT4 receptor agonist)

**Table 6:** Management of urinary dysfunction in PSP/CBS.

1. Pharmacologic therapy for overactive bladder
a. Alpha-receptor antagonists
i. Terazosin
ii. Doxazosin
iii. Tamsulosin
iv. Alfuzosin
v. Silodosin
b. 5-alpha reductase inhibitors
i. Finasteride
ii. Dutasteride
c. Beta-3 adrenoceptor agonists
i. Mirabegron
ii. Vibegron
d. Selective M3 anti-muscarinic anticholinergics
i. Darifenacin
ii. Solifenacin
e. Avoid non-selective anti-muscarinic agents due to central anticholinergic side effects
i. Avoid oxybutynin
ii. Avoid tolterodine
iii. Avoid fesoterodine
iv. Trospium has limited brain penetration and may be considered
f. BoNT injections for refractory overactive bladder
2. Non-pharmacologic therapy
a. Alcohol and caffeine avoidance
b. Nocturia
i. Compression stockings during day
ii. Elevate lower limbs in the late afternoon
iii. Restrict fluids in the evening
iv. Fully empty bladder before bed
v. Elevate the head of the bed at night
vi Bladder training and pelvic floor exercises
c. Condom catheter for men
d. Clean intermittent catheterization for refractory urinary voiding dysfunction with residual volume over 100 mL.
e. Trans-tibial nerve stimulation

Initial treatment of constipation centers on non-pharmacological interventions such as increased physical activity, hydration, and fiber supplementation. Increased water intake is necessary for the osmotic action of fiber, stool softeners, and laxatives in the gut. People with PSP and constipation should be counseled to drink 2 l of water per day. Of note, excessive hydration may increase the need to urinate, which can result in falls if PSP/CBS patients attempt to rush to the restroom unassisted. Psyllium, a form of fiber, increases bowel movement size, and frequency (102, 103).

The first-line pharmacologic agent is a stool softener such as docusate, starting at 100 mg daily, titrating to 100 mg TID. At least 5 days should be allowed between titration steps. Osmotic laxatives, particularly polyethylene glycol, are effective in Parkinson's disease (104) and recommended in PSP/CBS. Magnesium citrate, bisacodyl, senna, and lactulose can be considered as well. Magnesium citrate and lactulose are other useful osmotic agents in this setting. Bowel stimulants such as bisacodyl and senna should be used sparingly and for very brief periods. Prescription bowel stimulants such as linaclotide (a guanylate cyclase C agonist) and prucalopride (a selective 5-HT_4_ receptor agonist) were found to be safe and effective for the treatment of constipation in a small cohort of patients with Parkinsonism, including four individuals with PSP (105). These agents should be reserved for occasional use or refractory cases because their long-term safety has not been adequately evaluated.

### Evaluation of Urinary Symptoms

Urinary symptoms affect 53–93% of people with PSP (97, 98, 106), and early onset confers poorer disease prognosis (97). The most common urinary symptoms in PSP include urinary urgency, incontinence, and hesitancy (107). Detrusor overactivity is by far the most common symptom, producing frequency, urgency, and urge incontinence. Urodynamic testing may be critical in establishing a diagnosis and guiding pharmacologic treatment, especially in men (108, 109). Non-bladder-related contributions to urinary incontinence in PSP could be behavioral disinhibition, apathy, communication difficulty, and mobility problems (99, 100, 110). Before initiating treatment, it is important to review concurrent medications to identify drugs that may influence urinary symptoms, such as diuretics, anticholinesterases (for dementia) or anticholinergics, and to rule out prostate hypertrophy (111). A bladder diary may be helpful in providing data for the physician's visit (112). A proper abdominal, pelvic, and/or prostate exam to rule out non-neurological causes of incontinence or retention should be done. Workup may include urinalysis, electrolytes, BUN, creatinine, PSA, and bladder ultrasound. It is important to clarify the cause of symptoms, keeping in mind that in people with PSP, the clinical complaints poorly match the results of urodynamic studies (113).

### Management of Urinary Symptoms

Behavioral modifications such as alcohol and caffeine avoidance are first-line treatment for overactive bladder (111). Nocturia can be improved by using compression stockings during the day, elevating the lower limbs in the late afternoon, restricting fluids in the evening, fully emptying the bladder before bed, and elevating the head of the bed to reduce renal perfusion (114). Bladder training (115) and pelvic floor exercises using biofeedback (116) with pelvic therapy may improve symptoms but may be difficult for PSP/CBS patients to perform.

Alpha-receptor antagonists (i.e., terazosin, doxazosin, tamsulosin, alfuzosin, silodosin) are first-line pharmacological agents for treatment of bladder outlet obstruction and work by relaxing smooth muscle at the bladder neck and prostate. Of note, orthostatic hypotension is a potential concern in PSP/CBS with these agents. 5-alpha reductase inhibitors such as finasteride and dutasteride, typically used for prostate enlargement, can improve urinary dysfunction in men (117, 118). Non-selective anti-muscarinic anticholinergic agents (oxybutynin, tolterodine, fesoterodine) are used to treat urge incontinence but should be avoided in PSP/CBS due to their central anticholinergic side effects. Darifenacin and solifenacin, as selective M3 anti-muscarinics, have fewer side effects than traditional anticholinergics. Solifenacin is shown to be beneficial in incontinence in PD and may thus be useful in PSP/CBS as well, although a trial in PSP or CBS has not been performed (119). Trospium, a non-selective anti-muscarinic, has limited blood-brain barrier penetration (120), which may reduce CNS side effects. Beta-3 adrenoceptor agonists such as mirabegron are effective alternatives to the anticholinergic class. One small study showed mirabegron is safe and effective in PD with dementia (121) but, like the other agents, its efficacy and toxicity in PSP/CBS have not been adequately evaluated.

When urinary voiding dysfunction is present and refractory to pharmacologic intervention, clean intermittent catheterization when the postvoid residual is consistently above 100 ml is recommended (122). Condom catheters can be used in male patients to reduce caregiver strain and improve sleep. This may present unique challenges in people with advanced PSP or CBS with deficits in fine or gross motor control and cognition. Intermittent catheterization training should be completed with the patient and a caregiver and may be reinforced with pelvic floor-strengthening therapy to help with positioning and safety. Botulinum toxin injections for overactive bladder can also be helpful, but a potential side effect is urinary retention. Trans-tibial nerve stimulation may be helpful in decreasing urgency and frequency as well.

## Sleep Disturbances

Disordered sleep has been an under-recognized feature of PSP (Table 7). The sleep issues in PSP are distressing, often progressive, and are a frequent cause of impaired daytime functioning (123). Phenomena such as insomnia, excessive daytime sleepiness (EDS) and sleep fragmentation are common in neurodegenerative disorders generally and PSP/CBS specifically (124). Severe insomnia becomes common as the disease progresses (124) and patients with PSP frequently have sleep disordered breathing (125, 126). In PSP, EDS often occurs without night-time insomnia, suggesting a primary hypersomnia, but without features of narcolepsy (126). The prevalence of RBD in patients with PSP ranges from 14 to 33%, but it is not clear if this exceeds that in the general population after correcting for the effects of concomitant medications. It is clear, however, that the RBD frequency in PSP is less than for PD, where it ranges up to 60% (125–127). In PSP, unlike in PD, RBD does not typically predate onset of motor symptoms and physiologically documented REM without atonia may occur in PSP without clinical RBD complaints (126). Overnight polysomnography is the gold standard for diagnosing several sleep disorders and should be considered for PSP/CBS patients with notable sleep complaints that are not resolved with sleep hygiene improvements and perhaps a bedtime sedative.

**Table 7:** Management of sleep disturbances in PSP/CBS.

1. Pharmacologic therapy for insomnia
a. Melatonin (3–18 mg nightly)
b. Low dose clonazepam (0.25–1 mg nightly)
c. Other benzodiazepines, benzodiazepine receptor agonists
i. Zolpidem
ii. Eszopiclone
iii. OTC sleep aids such as diphenhydramine and others
d. Atypical and tricyclic antidepressants should be avoided or used with extreme caution
2. Pharmacologic therapy for excessive daytime somnolence
a. Stimulants such as modafinil, armodafinil, or methylphenidate
b. Use these at low dosages
3. Pharmacologic therapy for RLS/PLMD
a. Iron replacement (ferrous sulfate 325 mg daily with Vitamin C) if serum ferritin <75 mcg/l
b. Gabapentin enacarbil
c. Pregabalin
d. Dopamine agonists
i. Pramipexole
ii. Ropinirole
iii. Rotigotine
iv. Cabergoline
4. Pharmacological therapy for REM behavioral disorder
a. Melatonin (3–18 mg nightly)
b. Low dose clonazepam (0.25–1 mg nightly)
c. Gabapentin (100–300 mg nightly)
5. Non-pharmacologic therapy for insomnia
a. Circadian alignment
b. Sleep hygiene
c. Treatment of underlying issues
i. Depression
ii. Immobility
iii. Nocturia
6. Non-pharmacologic therapy for obstructive sleep apnea
a. Lateral decubitus positioning during sleep
b. Weight loss in the setting of obesity
c. Elevating the head of the bed
d. Splinting with oral appliances
e. Continuous positive airway pressure
f. Surgical removal of obstructive tissue
g. Activation of tongue protrusion by implantation of a hypoglossal nerve stimulator

Insomnia is a failure to successfully initiate or maintain restful sleep. In PSP, insomnia may be a result of difficulty turning in bed and urinary urgency/nocturia (128). Therefore, addressing these underlying issues by treating depression or detrusor hyperactivity may substantially improve insomnia. Strategies include circadian alignment, melatonin and if necessary, sedating medications (129).

Despite case reports of zolpidem giving improvement in sleep, motor, and other symptoms in isolated PSP patients, rigorous long-term studies are lacking (130). Benzodiazepines (clonazepam, temazepam) and benzodiazepine receptor agonists (zolpidem, eszopiclone) should be used with great caution in PSP/CBS given the risk of falls associated with these conditions (131–134). Although trazodone and mirtazapine are used frequently for insomnia in PSP/CBS, there is no evidence to support the use of these or other atypical antidepressants for this indication in the absence of depression (135). Tricyclic antidepressants such as doxepin or nortriptyline at low doses can be considered for insomnia in PSP/CBS but are limited by central anticholinergic side effects and the potential worsening in balance and increased fall risk. Over-the-counter sleep aids—most notably, diphenhydramine—should be avoided due to cognitive adverse effects in older adults (136). An ongoing randomized controlled trial is comparing the efficacy of zolpidem vs. suvorexant (an antagonist of the hypothalamic neuropeptide orexin) vs. placebo in individuals with PSP (ClinicalTrials.gov NCT04014387).

Excessive daytime sleepiness is common and distressing in PSP/CBS (137) and often does not improve with successful treatment of nighttime insomnia. Modafinil, armodafinil, or methylphenidate can be considered to treat this symptom (138, 139).

Clinicians should also evaluate for sleep fragmentation disorders, including obstructive sleep apnea (OSA), periodic limb movement disorder (PLMD), and restless legs syndrome (RLS). OSA and PLMD affect ~32 and 53% of individuals with PSP, respectively (140). RLS has been reported in 57% of PSP patients and is associated with reduced sleep duration and efficiency (141). OSA may be effectively treated by splinting with oral appliances, continuous positive airway pressure, surgical removal of obstructive tissue, or activation of tongue protrusion by implantation of a hypoglossal nerve stimulator (142). Additional conservative approaches include lateral decubitus positioning during sleep, weight loss in the setting of obesity and elevating the head of the bed. Either of the last two may be instituted after a sleep study provides evidence for their efficacy. Lateral decubitus positioning may be enforced by inserting a tennis ball in a pocket in the center of the back of a t-shirt or by the more expensive method of a small positioning monitor strapped to the dorsal neck alerting the wearer to departures from the lateral decubitus position (Night Shift™ or others).

The treatment approaches for PLMD and RLS are similar to those for OSA. PLMD during sleep without insomnia or EDS may not require treatment. Serum iron studies should be checked in all patients with RLS and a trial of oral iron therapy (ferrous sulfate 325 mg daily with Vitamin C) given to patients with iron deficiency or ferritin levels 75 mcg/l or lower (143). Gabapentin enacarbil, pregabalin, pramipexole, ropinirole, rotigotine, and cabergoline have moderate to strong evidence levels of benefit in RLS and PLMD (143), but these medications should be used with caution in PSP/CBS to avoid sedation and psychosis as side effects.

Treatment can be initiated for RBD on the basis of clinical history provided by the bed partner, but the possibility of nighttime psychosis must be kept in mind. The primary goal of treatment is to decrease the risk for sleep-related injury, including the removal of weapons and sharp-edged furniture from the bedroom. Oral treatment options include melatonin (3–18 mg) nightly (144) or low doses of clonazepam (0.25–1.0 mg) (145). While clonazepam is considered first line treatment for RBD, melatonin is often effective and better tolerated (144).

In summary, sleep disturbances in PSP are frequent and may severely impair quality of life. Overnight polysomnography should be considered to evaluate underlying causes of sleep disturbance. Several non-pharmacological and pharmacological treatment options exist, although evidence specific to PSP/CBS-related sleep dysfunction is sparse.

## Sialorrhea and Dysphagia

### Sialorrhea

Sialorrhea (excessive pooling of saliva and drooling) is common in Parkinsonian disorders including PSP/CBS and PD (Table 8). Insufficient automatic swallowing and complications of cholinergic or neuroleptic medications are the major contributors to sialorrhea (146).

**Table 8:** Management of sialorrhea and dysphagia in PSP/CBS.

1. Pharmacologic therapy for sialorrhea
a. BoNT injections are first line therapy
b. Oral glycopyrrolate (monitor for anticholinergic effects)
c. Sublingual atropine drops not recommended because of central anticholinergic effects
2. Non-pharmacologic therapy for dysphagia
a. Conservative measures
i. Postural maneuvers (chin-tuck posture, head turns)
ii. Alternating small bites and sips
iii. Taking multiple swallows
iv. Volitional coughing and throat-clears after swallowing
v. Breath-hold or supraglottic swallow
vi. As suggested by results of modified barium swallow
1. Thickening liquids to nectar or honey-thick consistency
2. Avoiding dry solids or mixed consistencies
b. Early speech language pathology referral
c. Tube feedings/percutaneous gastrostomy
i. Reserve for severe cases
ii. Carefully consider ethical and quality-of-life issues.

The standard treatment for sialorrhea in PSP/CBS is botulinum toxin injections, with oral anticholinergic therapy used typically as a bridge in cases where injections wear off before the next dose or where botulinum toxin injections are not feasible. Local botulinum toxin injections are the most effective treatment to reduce sialorrhea (147) and are initiated into the parotid glands. Submandibular glands may also be injected with good effect, but caution is advised due to their proximity to muscles of swallowing. The doses recommended by the manufacturers are 500–1,500 units of rimabotulinumtoxinB in each parotid gland and 250 units in each submandibular gland (148); 30 units of onabotulinumtoxinA and incobotulinumtoxinA per each parotid gland and 20 per each submandibular gland (149); and 15–75 units of abobotulinumtoxinA in each parotid and submandibular glands (150). Many experts agree that in refractory cases, higher doses may be effective without causing side effects. A review on efficacy in treating all-cause sialorrhea suggests level A evidence of benefit for rimabotulinumtoxinB (Myobloc) and level B evidence for onabotulinumtoxinA (Botox) and abobotulinumtoxinA (Dysport) (151). Myobloc and IncobotulinumtoxinA (Xeomin) currently have FDA approval for treatment of sialorrhea.

The main side effects of salivary gland botulinum toxin injections are dry mouth and worsening of dysphagia. This is of special concern in patients with PSP or CBS and use of botulinum toxin into structures of the lower jaw and neck in patients with more than mild dysphagia is not recommended.

Radiation therapy to the parotid and submandibular glands can also improve sialorrhea, with transient loss of taste and dry mouth (152); this is not routinely recommended for PSP/CBS. Some patients with PSP/CBS find that sucking on a lollipop stimulates the swallowing reflex, often reducing drooling despite increased production of saliva. Blocking cholinergic (particularly muscarinic M3) receptors can minimize salivary secretion, but at the cost of central and peripheral anticholinergic side effects. The benefit/side effect ratio of oral glycopyrrolate makes it the preferred anticholinergic for sialorrhea in PSP/CBS. Sublingual atropine 1% solution and amitriptyline, hyoscyamine, and transdermal scopolamine may be equally effective but typically have more side effects (146, 147, 153) such as confusion, hallucinations, drowsiness, constipation, and urinary retention.

### Dysphagia

Dysphagia (swallowing difficulty) impairs the safety and efficiency of oral intake and is a key source of morbidity and mortality in PSP/CBS. Dysphagia can impair nutrition, hydration and medication administration, and may cause death through aspiration pneumonia. Frank airway obstruction by a bolus of solid food is uncommon.

Dysphagia has a later median onset latency in CBS than in PSP (64 vs. 42 months after symptom onset, respectively) (154). Shorter latency from the onset of the PSP symptoms to development of dysphagia is associated with worse overall prognosis (154–157).

Patients with PSP/CBS often have impairments of both the oral and pharyngeal phases of swallowing. Signs and symptoms associated with dysphagia in PSP can include delayed swallow initiation, disorganized, or hyperkinetic tongue movements, slowing of the oral phase, prolonged mastication and poor transfer of the bolus to the pharynx (158, 159). Additional deficits include sialorrhea, vallecular pooling, incomplete epiglottic descent, and other physiologic abnormalities. These permit fluids or chewed food to drop into the pharynx and are associated with prolonged mealtimes, fatigue, oral cavity residue, nasal regurgitation, wet-sounding vocal quality, coughing, throat-clearing, and choking during oral intake. Increased vocal cord bowing caused by muscular atrophy was recently identified as a deficit in PSP that impairs cough efficiency and leads to aspiration (160). On the other hand, “piecemeal deglutition” (excessive lingual gestures causing multiple swallows required to clear a single bolus) is characteristic of CBS patients as assessed by Modified Barium Swallow (MBS) testing (161). Studies on dysphagia in CBS are sparse and until that changes, the principles of assessment and management of dysphagia in PSP also apply to CBS.

Patient-reported measures can evaluate dysphagia and include the Swallowing Quality of Life Questionnaire, the Stanford Swallowing Disturbance Questionnaire, the EAT-10 Tool, and the NIH-Speech Pathology swallowing questionnaire (161–163). The MGH Swallow Screening Tool and the Yale Swallow Protocol are useful bedside evaluations administered by the speech-language pathologist SLP (161, 162). The PSP Rating Scale includes items assessing solid swallowing by history and liquid swallowing by examination (41). A recently developed PSP Clinical Deficits Scale also captures elements of dysphagia (164). Once dysphagia is suspected, typically signified by coughing while drinking liquids, a referral should be made to a speech-language pathologist SLP. The SLP performs a clinical swallow evaluation consisting of a speech swallowing questionnaire, oral motor examination, and administration of oral trials. As the clinical swallow evaluation alone cannot rule out silent aspiration, which is common in PSP (158), the SLP usually requires instrumental assessments, even at the initial evaluation. A MBS with videofluoroscopy using a range of food and drink textures is performed as a routine part of an initial swallowing evaluation (165). This is “modified” to distinguish it from an ordinary barium swallow, which evaluates the esophagus but not the oropharynx. The MBS may be supplemented in selected cases by flexible (or fiberoptic) endoscopic evaluations of swallowing.

Levodopa and anticholinergic medications do not typically benefit dysphagia in PSP or CBS (158, 166). However, for levodopa-responsive patients with PSP-Parkinsonism, dosing levodopa approximately half an hour before mealtimes may modestly aid feeding and swallowing (158). There are no available studies formally assessing the impact of speech therapy on dysphagia in PSP patients, but benefit for dysphagia was demonstrated in PD patients undergoing Lee Silverman Voice Treatment (LSVT) (167, 168). We recommend early SLP referral for swallow therapy and regular reassessment of swallow function. Other non-pharmacological approaches the SLP may recommend in selected cases to improve swallowing safety include: (1) postural maneuvers (chin-tuck posture, head turns) to redirect bolus flow for airway protection and improved clearance, (2) alternating small bites and sips, (3) taking multiple swallows, (4) volitional coughs and throat-clears after swallowing, (5) use of a breath-hold or supraglottic swallow technique, (6) thickening liquids to nectar or honey thick consistency, and (7) diet consistency modifications such as mechanical soft or blended/pureed consistency. Patients with PSP often over-stuff their mouths and should be advised to employ a slow rate of intake and limit the size of bites and sips. They should sit fully upright during oral intake and remain so for 15–30 min afterward to reduce the risk of retrograde bolus flow and reflux. The MBS provides important guidance for the selection of these measures and it is important to reassess swallow function with MBS as soon as patients report worsening dysphagia. Patients can be advised to avoid mixed consistencies such as vegetable soup or dry cereal with milk, where both solids and liquids are presented together, and to take medications crushed and mixed in a pureed consistency such as apple sauce, yogurt, or pudding. Patients should avoid talking while eating and eating when tired. It is often useful to consult a dietitian to evaluate the adequacy of intake and nutritional status.

When even a modified diet becomes unsafe or oral intake no longer satisfies nutritional, hydration and medication administration needs, tube feedings should be considered. The discussion with patients and family should take quality of life into account, considering the patient's cognitive state and physical discomfort. Percutaneous gastrostomy should be considered when MBS shows aspiration or laryngeal penetration of all textures unimproved by feeding modification or therapy and either (a) loss of at least 10% body weight, (b) a first episode of aspiration pneumonia, (c) fever of unknown origin, or (d) mealtime duration of >1 h (169). It should be noted that feeding tube placement does not prevent the possibility of post-prandial aspiration of reflux or secretions but may mitigate prandial aspiration and improve nutrition and hydration status.

## Speech Disorders

Speech disorders are a common and often disabling manifestation of PSP and CBS (170) (Table 9). They may present early in the course and may progress to anarthria (170). They can be classified into three often-overlapping categories: dysarthria, a disturbance in neuromuscular control; apraxia of speech, the inability to generate motor programs for speech without loss of semantic skills; and non-fluent aphasia, the loss of language fluency with preservation of comprehension and semantic skills.

**Table 9:** Management of speech impairment in PSP/CBS.

1. Dysarthria
a. Speech therapy to maximize comprehensibility
b. Augmentative and alternative means of communications as disease progresses
i. Delayed auditory feedback or pacing boards
ii. Alphabet board
iii. Text-to-speech system
iv. Eye-gaze speech-generating devices
v. Eye-gaze speech-generating devices
2. Apraxia of speech
a. Intense, repetitive exercises
b. Metronome and other pacing methods
c. Gesture
d. Writing
e. Experimental: cerebellar repetitive transcranial magnetic stimulation
3. Non-fluent aphasia
a. Exercises to improve pace of speech
b. Gesture and other non-speech modalities
c. Caregiver and family determine topic and tone of conversation
d. Experimental: dorsolateral prefrontal cortical transcranial direct current stimulation
4. Palilalia
a. Pacing board
b. Metronome and other pacing methods

Evaluation of speech begins in the clinic by the physician and should only then proceed to the SLP. A standardized assessment of the impact of speech disorders is included as part of the PSP-Quality of Life scale (171). The frequent co-occurrence of speech disorders with dysphagia dictates a swallowing evaluation as a routine part of the SLP assessment. In selected cases, referral to ENT may be recommended to rule out anatomical pathology.

First, we recommend emphasizing the importance of strategies to maximize comprehensibility of the speech in context. These include securing the listener's attention and maintaining eye contact; avoiding distractions and multitasking; and ensuring that the topic of conversation is conveyed and understood at the beginning of the conversation. In addition, devices like delayed auditory feedback or pacing boards can help slow the speech rate, increasing comprehensibility (172).

### Dysarthria

Tools like the Mayo Clinic Motor Speech Disorders Rating Scale, Diadochokinetic Rate and the Frenchay Dysarthria Assessment can be helpful guiding the assessment of dysarthria (173–175), Management goals in the early disease stages focus on maintaining intelligibility of speech, followed by addition of augmentative and alternative means of communication with the progression of dysarthria. Speech therapy, particularly LSVT, which focuses on increasing loudness, may provide some benefit (176). Augmentative and alternative communication strategies include an alphabet board, a text-to-speech system, or eye-gaze speech-generating devices. These may be introduced initially to augment speech and later as a primary mode of communication (177).

### Apraxia of Speech

The Apraxia of Speech Rating Scale is a useful diagnostic tool (178). As for dysarthria, contextual strategies to improve information transfer play an important role.

Traditional approaches to treatment involve intense, repetitive exercises in order to practice the correct movement of muscles. Speech therapies may also include speaking paced by a metronome. Since Apraxia of speech is thought to be a disorder of coordinating the muscles of speech, augmentative therapies for communication may be useful, including the use of non-speech modalities such as gestures and writing. Non-invasive brain stimulation therapies such as cerebellar repetitive transcranial magnetic stimulation appear promising in anecdotal reports but require further studies in PSP/CBS (179).

### Palilalia

Palilalia is the involuntary repetition of syllables words or phrases, often with increasing pace. Unlike echolalia, palilalia involves repetition of one's own speech. Treatment may involve use of a pacing board or another method to pace oral production.

### Non-fluent Aphasia

Specific deficits comprising non-fluent aphasia in PSP/CBS include simplified or agrammatic speech produced in an effortful manner, with impaired grammatical comprehension and preserved lexical comprehension; and often accompanied by Apraxia of Speech. A version of the Screening for Aphasia in Neurodegeneration battery has been used for PSP patients and proposed as a reliable tool to screen for these characteristic language disturbances (180). For treatment of speech apraxia in PSP, one recent study (181) provides Class III evidence that transcranial direct current stimulation over the dorsolateral prefrontal cortex improves performance in some language tasks, but more study is required.

Traditional speech therapies often involve exercises to augment the pace or content of speech. Some speech therapies target specific aspects of impaired speech during therapy such as the production of verb phrases. Studies with small series of patients have reported an improvement in the non-fluent and agrammatic components of speech with the use of tDCS (179, 181). Augmentative therapies involve the use of non-speech modalities such as gesture to support communicative efficacy. Caregivers and loved ones can support communicative efficacy by establishing the topic of conversation and helping to mediate the nature of a conversational exchange.

## Cognitive and Behavioral Impairments

Behavioral and cognitive symptoms are the presenting feature in ~20% of PSP patients (182) (Tables 10, 11). The principal cognitive impairments of PSP affect abstract thought and verbal fluency, while forgetfulness and visuospatial issues are mild to moderate until advanced disease stages. In contrast, CBS patients exhibit predominantly ideomotor apraxia with attentional and visuospatial deficits, but executive function is less impaired overall than in PSP (182).

**Table 10:** Management of cognitive impairment in PSP/CBS.

1. Pharmacological management
a. Cholinesterase inhibitors such as donepezil
i. Use only for amnestic deficits
ii. Avoid in cases of severe postural instability
iii. Discontinue if no observed benefit
2. Non-pharmacological management
a. Caregiver and family education
b. Maintain a daily routine
i. Adapt previous activities to reduced abilities
ii. Emphasize enjoyable, rather than therapeutic, activities
iii. Minimize distractors
iv. Occupational therapy
1. Safety screening
a. Medication management
b. Financial risk
2. Access to stove, car, firearms
c. Compensatory technology
1. Daily planners
2. Mobile devices
d. Lifestyle modifications
1. Aerobic exercise
2. Heart-healthy diet
3. Adequate sleep and nutrition
4. Staying cognitively and socially engaged

**Table 11:** Management of behavioral disturbances in PSP/CBS.

1. Pharmacologic management
a. Apathy:
i. Trial of modafinil or methylphenidate
b. Impulsivity
i. Behavioral approaches difficult because of frontal deficit.
ii. Reduce levodopa and other dopaminergic medications
iii. Trial of SSRI such as escitalopram or sertraline at standard dosages
iv. Mood stabilizers such as valproic acid and lamotrigine
v. ADHD drugs such as atomoxetine
c. Depression/Mood disorder
i Trial of SSRI at standard dosages
ii Avoid tricyclic antidepressants due to side effects
d. Pseudobulbar affect
i. Trial of SSRI at standard dosages
ii. Dextromethorphan-quinidine (second-line due to cost)
2. Non-pharmacologic management
a. Caregiver/family education emphasizing that deficit is part of disease
b. Occupational therapy
c. Cognitive behavioral therapy
d. Safety screening
i. Risk of medication mismanagement,
ii. Financial risk
iii. Use of a stove
iv. Driving
v. Exposure to unsecured firearms

The prevalence of executive dysfunction can be as high as 30% in the pre-diagnostic phase of PSP, and others have demonstrated this disturbance early in the disease course in more than 70% of patients (183, 184). The memory disturbance in PSP and CBS is usually related to executive dysfunction and is characterized by deficits in retrieval with relatively preserved encoding (learning new information as tested by cued retrieval). PSP cases that present similarly to behavioral-variant FTD (chiefly behavioral apathy) are known as PSP- frontal variant according to the current PSP criteria (3). Both forms feature executive dysfunction. Impulsivity, disinhibition and at times lack of insight may also occur in both, bvFTD as well as PSP-RS, but in bvFTD, these deficits are expressed in the patient's behavior toward others, often in the form of taboo expression or action, while in PSP-RS, they are more directed toward their own postural instability and fall risk. Prevalence of PSP-F among PSP cases is estimated at 4.5 to 12% (180, 185). Many such patients require careful questioning and formal neuropsychological testing (186), but the Frontal Assessment Battery (FAB) (particularly its lexical fluency and Luria motor sequencing items), is a brief and practical tool for capturing subtle frontal impairments (187). Other executive function assessment tools include the FTD subscale of Clinical Dementia Rating Scale (CDR)-Sum of Boxes and several items of the MoCA. Some patients presenting with frontal executive deficits are mislabeled as having FTD, or rarely Alzheimer's disease (AD) (182). Screening tools like the FAB and MoCA can help assess for executive or memory impairment and are recommended for all patients. The more time-consuming Addenbrooke's Cognitive Examination Revised (ACE-III) is more a sensitive tool to capture the poor executive, episodic memory and visuospatial functions of PSP and can be considered as well (188). If screening is inconclusive, a complete neuropsychological evaluation is indicated to assess performance in other cognitive domains (182, 189, 190).

Distinguishing apathy from depression may affect management by helping avoid futile trials of antidepressants. Unfortunately, there are no effective treatments for apathy. It is extremely important to educate the family to understand that the behavior is part of the disease and not lack of interest in their loved ones. There are no relevant pharmacological trials, but in practice some physicians use modafinil or methylphenidate with unclear benefit.

For patients who present with encoding impairments, AD co-pathology should be considered. Drawing or copying spatially complex or three-dimensional figures evaluates planning, visual perception, and general cognitive control processes and provides qualitative characterization of the deficit—for example, in distinguishing planning deficits from spatial deficits. There are many coding systems available for this purpose, such as the Rey or Rey-Osterrieth Complex Figure Test (191).

For all of the cognitive and behavioral aspects of PSP/CBS, caregiver education is paramount. Suitable material and educational programs are available from reputable lay support organizations such as CurePSP, the PSP Association of the UK, and the Association for Frontotemporal Dementia.

Despite some symptom overlap between FTD, AD, and PSP, pharmacological treatment of cognitive loss in PSP is different from that of the others. A trial of a cholinesterase inhibitor may be justifiable if cognitive symptoms similar to those observed in AD (impaired memory encoding, not simply impaired recall with intact recognition memory) predominate, as cholinergic neuron loss is documented in PSP (189). Cholinesterase inhibitors are not recommended if severe motor features are present, as any positive effects on memory may be negated by worsening of postural instability (15). Similarly, the anti-glutamatergic drug memantine may worsen cognition in PSP and CBS and is also not recommended (192–194). Modafinil may improve apathy in some patients (190). Impulsive behavior may be addressed by reducing levodopa and other dopaminergic medications. Benzodiazepines for agitation should be used with caution and at low dosages because of their risk of exacerbating falls and confusion. Non-dopaminergic SSRIs (195) such as escitalopram and sertraline at standard dosages may be used, but these are often ineffective. Management of impulsivity is challenging if unrelated to specific medications that can be reduced or discontinued. Behavioral approaches are difficult to implement due to other frontal disturbances. Impulsivity is sometimes treated with mood stabilizers such as valproic acid and lamotrigine, or with medications for ADHD such as atomoxetine, but there are no trials showing efficacy in for these features, either in PSP/CBS or elsewhere.

Since executive dysfunction is exacerbated by affective lability, a SSRI may stabilize mood, provide antidepressant activity and ameliorate pseudobulbar affect. Dextromethorphan-quinidine may be used for pseudobulbar affect, but its cost renders it a second choice after SSRIs. Tricyclic antidepressants should be avoided due to anticholinergic side effects.

Non-pharmacological strategies for PSP and CBS include cognitive behavioral therapy, a daily routine, mobile devices and a daily planner, although none of these approaches has been formally tested. OT and education of patients and families on how to deal with executive dysfunction form the basis of management. OT includes safety screening (risk for medication mismanagement, financial risk, use of a stove, driving, access to unsecured firearms), environmental modifications (structured routine, minimized distractors, simplified tasks), and compensatory technology (daily planners, mobile devices) (196). Families should be informed that a false or exaggerated impression of dementia can be created by the patient's apathy, depression, dysarthria, fixed expression, and poor eye contact. General recommendations on lifestyle modifications for dementia are important. Examples include aerobic exercise, a heart-healthy diet, adequate sleep and nutrition, staying cognitively and socially engaged, simplifying the rules of games previously enjoyed by the patient to a level appropriate to their ability, (197). Games and other activities that interact with others and that are enjoyable, as opposed to educational or “therapeutic,” are especially recommended. It is paramount that the patient's caregivers understand that executive dysfunction and personality changes are part of the illness and that their impatience and frustration can aggravate the patient's existing affective state, which may reduce their quality of life. Caregivers are well-advised to try to anticipate and prevent errors rather than to correct them after the fact.

Further therapeutic approaches are urgently needed for the management of cognition in PSP/CBS (198).

## Limb Apraxia

Apraxia is defined as a deficit of motor programming that affects the performance of a learned skilled movement and is not caused by deficits in other cognitive, sensory, or motor domains (199) (Table 12). Limb apraxia is a well-known feature of CBS but is nearly as common in PSP-CBS and only slightly less frequent than that, although milder, in PSP-RS (200, 201). Studies evaluating the frequency of limb apraxia have found up to 57% in autopsy proven CBD, but this is likely underestimated (200).

**Table 12:** Management of limb apraxia in PSP/CBS.

1. Identify and address other factors impairing performance
a. Motor deficits
b. Cognitive deficits
c. Occupational therapy
i. Evaluate for environmental changes
ii. Simplify tasks to reduce the need for manual dexterity
iii. Gestural, direct and explorative training
iv. Encourage bimanual or bipedal tasks for asymmetric motor neglect or alien limb
v. Mirror therapy
vi. Repetitive facilitation exercise and video game-based rehabilitation

To aid the clinical evaluation of apraxia, several short screening tools appropriate for bedside testing have been developed, including the Apraxia Screen of the Test of Upper Limb Apraxia (TULIA) and the Short Test for Apraxia (202, 203). More comprehensive batteries can be considered as well, including the Tool for Oral and Limb Apraxia, the De Renzi Ideomotor Apraxia Test, the complete TULIA, and the Florida Apraxia Battery (204–207).

The management of apraxia involves identifying and addressing other factors impairing performance of tasks. These include motor abnormalities such as Parkinsonism or dystonia, and cognitive deficits such as impaired executive function. Occupational therapy should consider evaluation for environmental changes such as removing unsafe objects and simplifying tasks to reduce the need for manual dexterity. Rehabilitation strategies may be useful in early stages but typically do not provide sustained benefit. Strategies commonly used in post-stroke apraxia such as gestural, direct, and explorative training may be useful as well (208). Mirror therapy and forced bimanual or bipedal tasks, found to improve function in post stroke patients with limb apraxia (209), may also be helpful in CBS and PSP based on anecdotal experience. Similarly, benefits from repetitive facilitation exercise and video game-based rehabilitation have been reported in CBS, but additional evidence is needed (72, 210). Finally, transcranial direct current stimulation to the left parietal cortex appears to be a promising therapy for limb apraxia in CBS, but larger multicenter studies are needed (211).

## Telemedicine

When in-person visits are difficult or impractical because of distance, advanced disease, or infection precautions, video (telemedicine) visits offer an alternative solution and are being more frequently utilized among movement disorders specialists (212). While there is no published literature regarding the use of real-time synchronous videoconferencing for PSP/CBS, this tool has been used successfully for the management of PD (213–216). A CurePSP Care Guide is available to assist nursing interventions by phone (217).

Among patients, caregivers and long-term care staff, lack of knowledge about PSP/CBS has been identified as a major challenge (218). Telemedicine may afford access to movement disorders specialists in underserved areas. An initial assessment questionnaire focusing on diagnosis, information needs, ADL, mood, cognition, and palliative care is helpful to individualize the intervention (217). In lieu of or in addition to in-person visits, nurses or physicians can use telemedicine to educate on disease progression, prevention of falls, development of support systems, recognition of depression, the importance of home safety, and information on research studies. Telemedicine provides a unique opportunity to evaluate home safety and the effectiveness of assistive devices in the home environment. Telemedicine may be used to remove barriers to participation in PSP and CBS clinical trials, but this is limited by the lack of validated tools. While virtual administration of the PSP Rating Scale has not been directly compared to in-person assessment, video recordings of the scale that omitted direct examination of limb rigidity, dystonia and dysphagia were shown to have good inter-rater reliability with Cronbach's alpha = 0.87 (41, 219). A multidisciplinary telemedicine protocol specifically for advanced, immobile patients with PSP or CBS would be valuable.

## Advance Care Planning and Caregiver Burden

Palliative care is often associated with end-of-life care, but in the case of PSP/CBS, should be considered at the time of diagnosis. Referral delays may add unnecessary distress to the individual and loved ones as the condition progresses. Data from a recent randomized trial highlight the value of palliative care for improving quality of life in PSP (220) and establish the need for innovative, integrated palliative care models, especially in the early disease stages (221). However, a multitude of interacting patient- and clinician-level factors may delay palliative care referral in PSP/CBS (222).

The American Academy of Neurology recommends that clinicians discuss advance care planning within the first year following diagnosis of PD and we recommend the same for PSP/CBS (222). It is crucial to revisit the preferences, values, and choices throughout the illness trajectory (220, 222, 223). One retrospective study examined prevalence of advanced care planning among 49 individuals with atypical Parkinsonism (224). About 80% had a living will and were more likely than individuals with PD to have completed multiple forms of advance care planning (224). Common symptoms prompting inpatient palliative care unit admission among 38 patients with PSP and CBS were dysphagia in 59%, gait instability/falls in 52%, and spasticity/pain in 46% (225). The response rates to inpatient palliative treatment in that study were highly satisfactory, ranging from 11% for dysphagia to 52% for spasticity and pain.

Home health care agencies can offer intermittent, time-limited and problem-focused nursing care (226). Formal caregivers may be hired for assistance with ADLs (227), providing respite for caregivers and improving quality of life. However, this service is not covered by most medical insurance and is more expensive than most families can afford, at least in the US. Home-based medical care programs have yielded high-quality care while decreasing acute care utilization and the likelihood of admission to an institutional setting (228, 229). One pilot study demonstrated the feasibility of interdisciplinary home visits for individuals with PSP and CBS (230), with ongoing trials under way. For patients requiring more comprehensive care, nursing facilities remain the main option, although staff members often have little familiarity with PSP/CBS. There are no outcome data for patients with PSP or CBS who have been transferred to nursing facilities relative to outcomes in other settings. Although most people would prefer end-of-life care and death to occur at home, this often proves difficult (228, 231). The value of palliative care consultation throughout the course of the disease—rather than solely at the end of life—should not be underestimated. Hospice allows patients to have caregiver, social workers and RN support covered by Medicare/Medicaid. In the US, many patients with Parkinsonism lose access to outpatient care and die without hospice services once institutionalized (232).

There is a paucity of data regarding the desire for assisted suicide among people with Parkinsonism (233). Leading authorities in other life-limiting illnesses, especially cancer, advocate against the practice (234), which is illegal in most jurisdictions in the US. To mitigate demoralization and similar reactions prompting the desire for hastened death in PSP and CBS, clinicians should help patients identify a purpose to live for the future rather than solely focusing on symptom management (223). Experienced clinicians may choose to devote nearly an entire visit to this purpose. There are no data on the efficacy and safety of antidepressant medications in this setting for PSP/CBS.

PSP places a significant burden on caregivers' quality of life. While many studies use generic assessments such as the Zarit Burden Interview (235), these lack content validity related to PSP or CBS. More recently developed, the PQoL Carers Scale specifically measures quality of life in caregivers of individuals with atypical Parkinsonism (236). Supportive counseling may be provided to caregivers by social workers, psychologists, and other mental health professionals. Respite care services that pay professional caregivers, providing a break for the primary caregiver ranging from hours to days, are invaluable. Area agencies on aging, social workers, and foundations (such as CurePSP in the US and the PSP Association in the UK) can offer information and funding for respite care resources.

## Experimental Approaches and Clinical Trials

Advances in the basic science of tauopathies have yielded several promising approaches to neuroprotection in PSP/CBS (Table 13). However, several attempts to slow disease progression have failed to demonstrate efficacy in appropriately powered, randomized placebo-controlled trials. The first was riluzole, a medication approved for ALS with multiple proposed mechanisms of action (237). Another failed approach was davunetide, a neuroprotective protein that stabilizes microtubules (238). More recent trials have targeted pathological tau protein. One, tideglusib, reduces tau phosphorylation by inhibiting glycogen synthase kinase-3 (239). The other two are monoclonal antibodies directed against the N-terminal of tau (240–242). None of the trials showed clinical efficacy, although tideglusib slowed whole brain, cerebral, parietal and occipital atrophy on MRI (243). For CBS, failed pilot trials have tested valproate and lithium (each proposed to act via GSK-3 beta inhibition), TPI-287 (a microtubule stabilizer), and young plasma infusions (244–246). Suggested reasons for trial failure include recruitment of patients at an advanced stage of neurodegeneration, failure of antibodies to bind tau effectively, and failure of the antibodies to engage its target in the brain despite reduction of lumbar CSF tau.

**Table 13:** Experimental neuroprotective approaches and clinical trials for PSP/CBS.

1. Completed clinical trials (none has shown clinical efficacy)
a. PSP
i. Riluzole (multiple proposed mechanisms of action)
ii. Davunetide (microtubule stabilizer)
iii. Tideglusib (GSK-3 beta inhibition to reduce hyperphosphorylation)
iv. Monoclonal antibodies directed against N-terminal of tau
b. CBS
i. Valproate (GSK-3 beta inhibition)
ii. Lithium (GSK-3 beta inhibition)
iii. TPI-287 (microtubule stabilizer)
iv. Young plasma infusions
2. Novel neuroprotective approaches in or near clinical trials
a. RT001 (anti-lipid peroxidation agent)
b. UCB0107 (anti-tau antibody targeting the microtubule binding domain rather than the N-terminal)
c. ASN120290 (O-GlcNAcase inhibitor to inhibit removal of O-linked *N*-acetylglucosamine from tau)
d. AZP2006 (increases progranulin)
e. MP201(mitochondrial uncoupler)
f. Tolfenamic acid (NSAID and tau transcription factor Sp1 inhibitor)
g. Antisense oligonucleotides (reduce tau production)

A number of promising novel neuroprotective approaches entering trials for PSP in the near future are reviewed in detail elsewhere (242). Some of these trials have been delayed by the COVID-19 pandemic. These include RT001, an anti-lipid peroxidation agent; UCB0107, an anti-tau antibody targeting the microtubule binding domain rather than the N-terminal; ASN120290, an O-GlcNAcase inhibitor, which inhibits tau phosphorylation by preventing removal of another post-translational modification by n-acetyl glucosamine; AZP2006, an agent that increases progranulin (which possibly interacts with tau) production; MP201, a mitochondrial uncoupler; the NSAID tolfenamic acid and its analogs as inhibitors of the tau transcription factor Sp1; and several antisense oligonucleotides to reduce tau production. Recruitment into PSP and CBS clinical trials to date has been robust and the growing number of clinical trials in this area gives ample reason for optimism. Further development of biomarkers such as tau PET ligands and assays for CSF 4R tau promises more sensitive diagnosis of PSP, allowing inclusion of earlier-stage increase recruitment of PSP/CBS patients.

## Multidisciplinary Care

Multidisciplinary team care for PSP/CBS typically includes a neurologist, physical, occupational, and speech-swallow therapists, a social worker, and potentially a neuropsychologist and nurse, if available. The core multidisciplinary team includes an array of professionals working together to evaluate and educate patients and caregivers (226, 247). The movement disorder neurologist assumes overall care, can refer to community resources or other professionals as needed, supports team members with the design of intervention plans and medical advice, and discusses advance directives (217, 247). The physical therapist plays a key role in the evaluation of gait and balance, helping to prevent falls with assistive devices and exercise programs that focus on balance, flexibility, endurance, and eye movements (32, 39, 51, 248). The occupational therapist evaluates ADL, offers guidance regarding home modifications, recommends assistive devices, suggests compensatory strategies for limited eye movements, and assesses driving performance. It is often helpful to have physical and occupational therapy evaluate the patient together and formulate a joint management plan in the multidisciplinary setting. The speech-swallow therapist and nutritionist/dietitian often work together to evaluate and manage dysphagia. The speech-swallow therapist can offer advice regarding secure swallowing, texture modification, and compensatory strategies for communication (249), while the nutritionist/dietitian can monitor weight and plan calorie supplementation when necessary. The social worker refers to community resources and advises on assisted living facilities, advance directives, and palliative care. The neuropsychologist can offer an in-depth cognitive evaluation and help assess decision-making capacity.

Clerici et al. evaluated a multidisciplinary intensive rehabilitation treatment (MIRT) approach in 24 PSP patients (250). Their protocol comprised 20 h per week of inpatient physical therapy focused on aerobic conditioning, gait and balance, occupational therapy, and speech therapy. The average total PSP Rating Scale improvement in the MIRT group was 8 points, where the baseline score was 34 of 100. The typical worsening on this scale is 11 points per year for PSP-RS (41, 238). This preliminary study demonstrates the short-term benefits of intensive multidisciplinary rehabilitation in PSP (251), but this approach has not been tested against standard care, nor in the outpatient setting.

## Author Contributions

BB and AP: contributed equally to the conception, organization, data gathering, and writing and reviewing. IL: organization, data gathering, and writing and reviewing. LG: conception, administrative support, organization, data gathering, and writing and reviewing. All others: data gathering and writing and reviewing.

## Conflict of Interest

The authors declare that the research was conducted in the absence of any commercial or financial relationships that could be construed as a potential conflict of interest.
